# Efficacy of Tai Chi on lower limb function of Parkinson’s disease patients: A systematic review and meta-analysis

**DOI:** 10.3389/fnagi.2023.1096417

**Published:** 2023-02-01

**Authors:** Ping-an Zhu, Qi-qi Lu, Zhi-liang Li, Rong-liang Hu, Shu Xu, Lisa Brodersen, Yuan-xin Liu, Howe Liu, Xiao Bao

**Affiliations:** ^1^Department of Rehabilitation Medicine, Yuebei People’s Hospital, Shaoguan, China; ^2^Department of Rehabilitation Medicine, Jiangmen Central Hospital, Guangdong, Jiangmen, China; ^3^Department of Rehabilitation Medicine, Shaoguan Railway Hospital, Shaoguan, China; ^4^Physical Therapy Program, Allen College, Waterloo, IA, United States; ^5^College of Sports and Health Science, Xi’an Physical Education University, Xi’an, China

**Keywords:** Parkinson’s disease, motor function, balance, gait, Tai Chi

## Abstract

**Background:**

At present, the effect of Tai Chi (TC) on lower limb function in patients with Parkinson’s disease (PD) is controversial. Therefore, we conducted a meta-analysis on the influence of TC on lower limb function in PD patients.

**Methods:**

According to the PRISMA guidelines, seven databases were searched. Randomized controlled trials (RCTS) were selected and screened according to inclusion and exclusion criteria. We assessed the quality of the studies using the Cochrane Risk of Bias tool and then extracted the characteristics of the included studies. The random effect model was adopted, and heterogeneity was measured by *I*^2^ statistic.

**Results:**

A total of 441 articles were screened, and 10 high-quality RCTs were with a total of 532 patients with PD met Our inclusion criteria. Meta-analysis showed that compared To control groups TC improved several outcomes. TC significantly improved motor function (SMD = −0.70; 95% CI = −0.95, −0.45; *p* < 0.001; *I*^2^ = 35%), although The results were not statistically significant for The subgroup analysis of TC duration (SMD = −0.70; 95% CI = −0.95, −0.45; *p* = 0.88; *I*^2^ = 0%;). TC significantly improved balance function (SMD = 0.89; 95% CI = 0.51, 1.27; *p* < 0.001; *I*^2^ = 54%), functional walking capacity (SMD = −1.24; 95% CI = −2.40, −0.09; *p* = 0.04; *I*^2^ = 95%), and gait velocity (SMD = 0.48; 95% CI = −0.02, 0.94; *p* = 0.04; *I*^2^ = 78%), But Did Not improve endurance (SMD = 0.31; 95% CI = −0.12, 0.75; *p* = 0.16; *I*^2^ = 0%), step length (SMD = 0.01; 95% CI = −0.34, 0.37; *p* = 0.94; *I*^2^ = 29%), and cadence (SMD = 0.06; 95% CI = −0.25, 0.36; *p* = 0.70; *I*^2^ = 0%).

**Conclusion:**

TC has beneficial effects on motor function, balance function, functional walking ability, and gait velocity, but does not improve walking endurance, stride length, and cadence.

## Introduction

As the older adult population increases, the risk of Parkinson Disease (PD) increases accordingly. As a chronic disease, PD has a long course. In 2016, about 61 million people worldwide were affected by PD (Bloem et al., 2021). The incidence rate and prevalence of this disease have risen rapidly in the past 20 years due to reasons not yet fully understood (Dorsey et al., 2018; Deuschl et al., 2020). In U.S. around 50,000 individuals are diagnosed with PD each year and 0.5 million individuals have the disease (Marras et al., 2018). Due to the aging population, prevalence of PD is expected to rise to more than 1 million by 2030 (Marras et al., 2018), highlighting the need for optimization of care and management for PD patients.

PD is the second most prevalent neurodegenerative disease globally (Collaborators, 2017). Its cardinal motor features include tremor, bradykinesia, rigidity, and postural instability that frequently lead to abnormal limb or trunk postures, impaired gait, and decreased physical activity (Jankovic, 2008). As one of the most common causes of disability (Opara et al., 2017), the PD patients has brought great burden on the social and family level (Ransmayr, 2020). PD often affects lower limb motor function, such as balance, gait, and mobility (Bloem et al., 2001; Mak and Wong-Yu, 2019). Balance disorders can impair the patient’s quality of life and function, and greatly increase the risk of falls (Bloem et al., 2001). Furthermore, as the disease progresses, PD patients lose balance, resulting in gait disorders and limitations on basic and instrumental activities of daily living (Willemsen et al., 2000). The loss of mobility in Parkinson’s is strongly associated with decreased quality of life and freezing of gait (FOG). As one of the most common complications of PD, FOG (Xie et al., 2020) limits mobility and increases the risk of falls (Latt et al., 2009; Perez-Lloret et al., 2014).

Currently, there are medical and non-medical interventions for motor dysfunction in patients with PD. Medical interventions are most commonly pharmacological interventions (dopamine replacement therapy or dopamine receptor agonists; Obeso et al., 2010). However, the long-term effects of these drugs include side effects, and the patient’s motor function continues to deteriorate (Ahlskog and Muenter, 2001; Manson et al., 2012; Kim et al., 2019).

Non-medical interventions usually refer to mind–body exercise therapy, which has been shown to improve motor dysfunction in patients with PD (Earhart and Falvo, 2013; van der Kolk and King, 2013). As a model of mind–body exercise, Tai Chi (TC) is a mind–body exercise that uses body movement as well as breathing control to improve breathing ability, balance, and postural control and to reduce stress and anxiety in the elderly (Mansky et al., 2006; Lan et al., 2013).

Metabolomics studies suggest that the imbalance of metabolites and metabolic pathways in PD patients are mainly related to amino acid metabolism, energy metabolism, and neurotransmitter metabolism (Shao and Le, 2019). Studies have also suggested that TC can improve the metabolism of amino acids, energy, and neurotransmitters in PD patients (Li et al., 2022). This suggests that TC may improve PD motor symptoms by regulating metabolites. Zhou et al. (2018) evaluated the changes of brain metabolites and muscle energetics in the elderly after TC training through the joint detection of brain muscle magnetic resonance spectroscopy (MRS), and found that the N-acetylaspartate to creatine (NAA/Cr) ratios in the elderly after cingulate gyrus significantly increased, and the recovery time of leg muscle phosphate (PCr) significantly prolonged. A meta-analysis summarized the effects of TC on biomarkers, including inflammatory cytokines, oxidative stressors, and neurotrophic factors. The results showed that the anti-inflammatory effect of TC may be (1) mainly through reducing proinflammatory cytokines and proinflammatory cytokines, and a small amount through increasing anti-inflammatory cytokines; (2) In the antioxidative stress, it increases the antioxidative process and decreases the pre oxidative process; (3) Improve neural plasticity by promoting the production of BDNF and NAA; (4) Increase transcription factor NF-K β. Further reduce the production of inflammatory factors and prooxidants (Liu et al., 2022). Li et al. (2022) studied the effect of TC on PD motor symptoms using resting-state functional magnetic resonance imaging(fMRI) and found that long-term TC training could improve PD motor function, especially gait and balance. Potential mechanisms may include enhanced brain network function, reduced inflammation, improved amino acid metabolism, energy metabolism, and neurotransmitter metabolism, as well as reduced vulnerability to dopaminergic degeneration (Li et al., 2022).

In the past two decades, TC has been prescribed as a sensorimotor agility exercise program for patients with PD (King and Horak, 2009) to improve general function, balance, gait, quality of life, and mental health (King and Horak, 2009; Lan et al., 2013). Li et al. (2012) also reported significant improvements in gait speed, functional Range Tests (FRT), and unified Parkinson’s Disease Rating Scales (UPDRS) after TC exercise (48 times over 24 weeks). Furthermore, studies have shown that TC can improve the gait, balance, and functional activity of PD patients, suggesting that TC may be an effective and safe form of exercise (Hackney and Earhart, 2008). However, another study has provided different results. Choi et al. (2013) found that after 36 treatments, the TC group did not improve motor symptoms in PD patients compared to the control group. Vergara-Diaz et al. (2018) used TC training for 2 weeks and found no significant difference in UPDRS motor score and Timed Up and Go (TUG) compared to the control group (conventional care). Because these studies have yielded inconsistent results, the purpose of this meta-analysis was to investigate the effects of TC on lower limb motor function in patients with PD.

## Materials and methods

### Protocol and registration

This study was designed and conducted based on the Preferred Reporting Items for Systematic Reviews and Meta-analysis (PRISMA) guidelines (Page et al., 2021). The study was registered in Prospero (CRD42022334099).

### Search strategy

Following the 2020 PRISMA Statement (Page et al., 2021), a systematic search was performed in seven databases [PubMed, Cochrane Library, EMBASE, web of science, Ovid, Wanfang and China National Knowledge Infrastructure (CNKI)]. The search date was closed to June 8, 2022. The search strategy in the PubMed database was to search using Tai Chi (MeSh) matching the following combination of keywords: (Secondary Parkinson Disease) OR (Symptomatic Parkinson Disease) OR (Parkinsonism, Symptomatic) OR (Symptomatic Parkinsonism).

### Inclusion and exclusion criteria

Eligible studies met the following criteria: (1) Study population: All patients met the diagnostic criteria for Parkinson’s disease and were diagnosed with PD by clinicians, (2) Compared with the control group (No intervention or low-intensity exercise), the intervention model of the experimental group was TC, (3) Outcome measures were at least one of three (motor function, balance, and gait), (4) The type of study was a randomized controlled trial. (5) The language included in the study was English or Chinese. Studies that met the following criteria were excluded: (1) Cross randomized controlled trial, (2) Unable to extract detailed data in outcome indicators, and (3) Research on non-Chinese core journals.

Two experienced researchers (ZPA and LZL) independently evaluated the studies based on the above inclusion/exclusion criteria. They conducted a preliminary screening through the title and abstract of the article. Then, the included and uncertain studies are evaluated in full text. All differences were discussed and resolved by two researchers.

### Data extraction

Two researchers (HLL and XS) independently extracted data from each study, including (1) basic information about the study (author, year of publication, country of study, type of study), (2) study grouping information (age, number, and intervention model of experimental group and control group), (3) intervention plan (time, frequency), (4) outcome indicators (motor function, balance, and gait). Mean and standard deviations (SD) were used in the meta-analysis. The means and SDs of the main clinical results were obtained from the tables published in each study. All differences were discussed and resolved by two researchers.

### Data synthesis and statistical analysis

Date analysis used RevMan5.4.1 statistics software. Standardized mean difference (SMD) was calculated in the meta-analysis and 95% confidence intervals (CI) were evaluated using the Z-test. Heterogeneity of the result of studies was evaluated by computing Q test where a *p* value of <0.05 or *I*^2^ > 50% was considered high heterogeneity. To ensure the stability of the results and find the reasons for the high heterogeneity, sensitivity analysis and subgroup analysis were conducted. Due to high heterogeneity of included studies, a random effects model was used for analysis.

## Results

### Literature search and study characteristics

**Figure 1:**
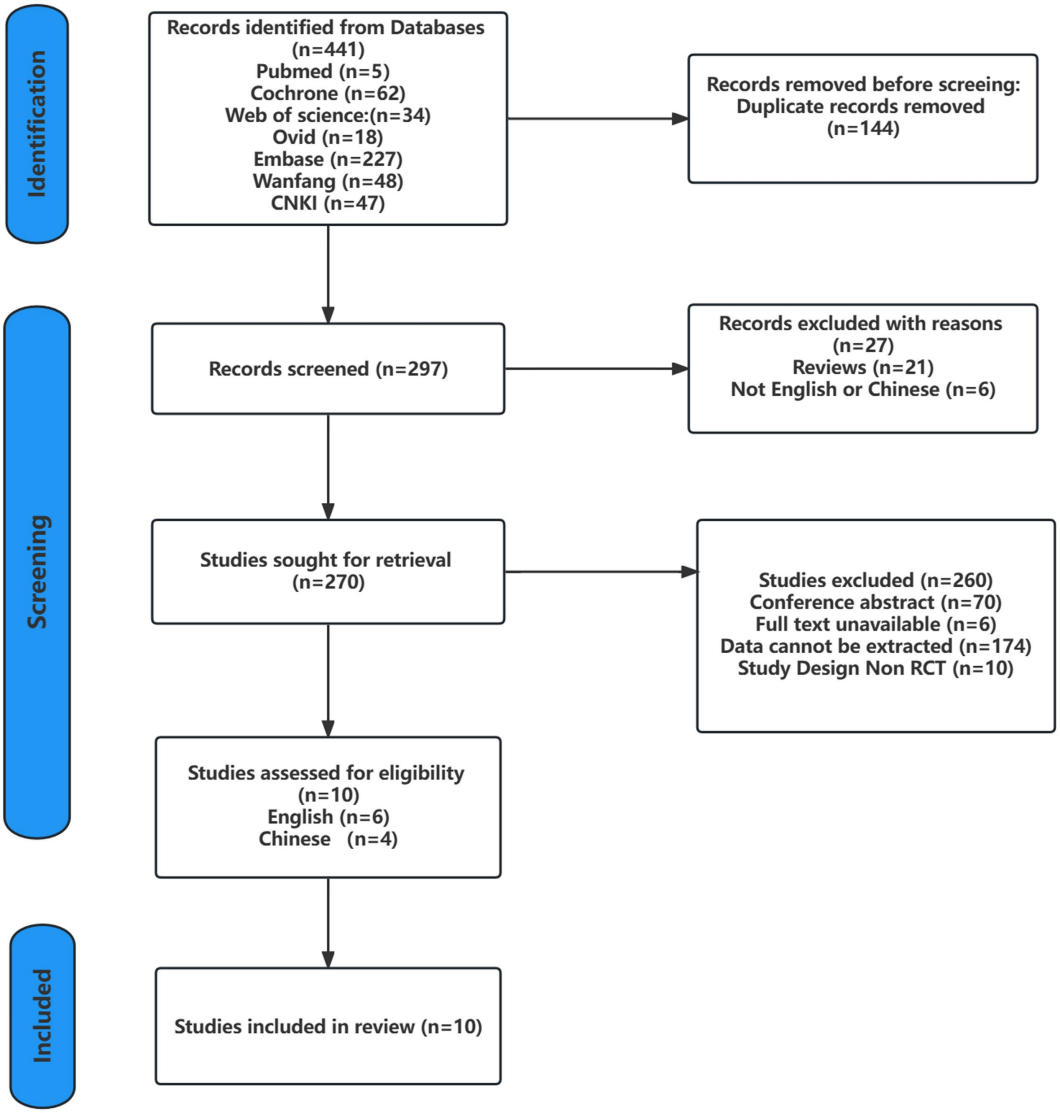
PRISMA flow chart of study selection.

Searches of the seven databases yielded 441 records. After screening against the inclusion criteria and excluding duplicate records, the relevant records were reduced to 291 articles, which were then screened by reading the titles, abstracts, and the full text, only 10 studies met the inclusion criteria. Six studies were written in English (Hackney and Earhart, 2008; Li et al., 2012, 2022; Amano et al., 2013; Choi et al., 2013; Vergara-Diaz et al., 2018), and four were written in Chinese (Yi et al., 2011; Suqiong et al., 2016; Li et al., 2017; Xihong et al., 2018). The detailed screening process is shown in Figure 1.

As shown in Table 1, the 10 selected studies represented 532 PD patients. The experimental group in each study received TC as an intervention, but TC styles were inconsistent. The TC style of five studies was Yang style (Hackney and Earhart, 2008; Yi et al., 2011; Amano et al., 2013; Li et al., 2017; Xihong et al., 2018), two studies were Chen style (Suqiong et al., 2016; Li et al., 2022), and the other three studies did not specify which TC style was. The control groups received no intervention or low-intensity exercise, such as walking and stretching. TC intervention time range is 1 to 12 months, nine studies (Hackney and Earhart, 2008; Yi et al., 2011; Li et al., 2012, 2017, 2022; Amano et al., 2013; Choi et al., 2013; Suqiong et al., 2016; Vergara-Diaz et al., 2018) used Unified Parkinson’s Disease Rating Scale motor score III (UPDRS III) to evaluate motor function in patients with Parkinson’s disease, five studies (Hackney and Earhart, 2008; Yi et al., 2011; Suqiong et al., 2016; Xihong et al., 2018; Li et al., 2022) used Berg balance Scale (BBS) to evaluate balance function, six studies (Hackney and Earhart, 2008; Li et al., 2012, 2022; Choi et al., 2013; Vergara-Diaz et al., 2018; Xihong et al., 2018) used timed up and Go (TUG) to evaluate functional walking ability. Three studies (Hackney and Earhart, 2008; Choi et al., 2013;Suqiong et al., 2016) used the 6-min walk test (6MWT) to assess walking endurance, stride length (four studies; Hackney and Earhart, 2008; Amano et al., 2013; Xihong et al., 2018; Li et al., 2022), cadence (three studies; Amano et al., 2013; Li et al., 2017, 2022), and gait velocity (six studies; Hackney and Earhart, 2008; Li et al., 2012, 2017, 2022; Amano et al., 2013; Xihong et al., 2018) to assess gait.

**Table 1:** Characteristics of the included studies.

Author (year)	Country	Study design	Age (years; M ± SD)	Experimental group (*N*)	Control group (*N*)	group	Intervention Protocol	Outcome
Amano et al. (2013)	US	RCT	Experimental group: 66 ± 11 Control group: 66 ± 12	15	9	Experimental group: TC Control group: no intervention	16 weeks; 3 times/week, 60 min/per time	Gait, UPDRS-III
Choi et al. (2013)	US	RCT	Experimental group: 60.81 ± 7.6 Control group: 65.54 ± 6.8	11	9	Experimental group: TC Control group: no intervention	12 week, 60 min/ per time	UPDRS-III, TUG, 6MWT
Hackney and Earhart (2008)	US	RCT	Experimental group: 64.9 ± 8.3 Control group: 62.6 ± 10.2	13	13	Experimental group: TC Control group: no intervention	13 weeks, twice a week，60 min/ per time	Gait, UPDRS-III, TUG, 6MWT, BBS
Li et al. (2012)	US	RCT	Experimental group:68 ± 9 Control group: 62.6 ± 10.2	65	65	Experimental group: TC Control group: Stretching	24 weeks, 2 times/week; 60 min/per time	Gait, UPDRS-III, TUG
Vergara-Diaz et al. (2018)	US	RCT	Experimental group: 65.7 ± 3.86 Control group: 62 ± 7.77	16	16	Experimental group: TC Control group: Usual Care	6 months, Twice a week, 60 min per time	UPDRS-III, TUG
Li et al. (2022)	US	RCT	Experimental group: 62.7 ± 5.51 Control group: 61.9 ± 6.76	32	32	Experimental group: TC Control group: no intervention	12 months, twice a week, 60 min per time	Gait, UPDRS-III TUG, BBS
Zhu et al. (2011)	China	RCT	Experimental group: 63.35 ± 8.72 Control group: 64.83 ± 9.29	19	19	Experimental group: TC Control group: walking	4 weeks, 10 times/week; 40 min/per time	UPDRS-III, BBS
Li et al. (2017)	China	RCT	Experimental group: 65.25 ± 6.37 Control group: 65.78 ± 5.36	42	38	Experimental group: TC Control group: no intervention	16 weeks, 3 times/week; 60 min/per time	Gait, UPDRS-III
Ji et al. (2016)	China	RCT	Experimental group: 56.06 ± 11.16 Control group: 59.13 ± 11.22	19	19	Experimental group: TC Control group: no intervention	24 weeks, 1 time/week 60 min/per time	UPDRS-III, 6MWT, BBS
Guan et al. (2018)	China	RCT	Experimental group: 69.46 ± 5.45 Control group: 68.61 ± 6. 22	40	40	Experimental group: TC Control group: no intervention	24 weeks, 4 times/week 60 min/per time	Gait, TUG, BBS

RCT, Randomized controlled trial; TC, Tai Chi; UPDRS-III, Unified Parkinson’s Disease Rating Scale Part III; TUG, timed up and go; BBS, berg balance scale; 6MWT, 6-min walk test.

### Quality assessment

**Figure 2:**
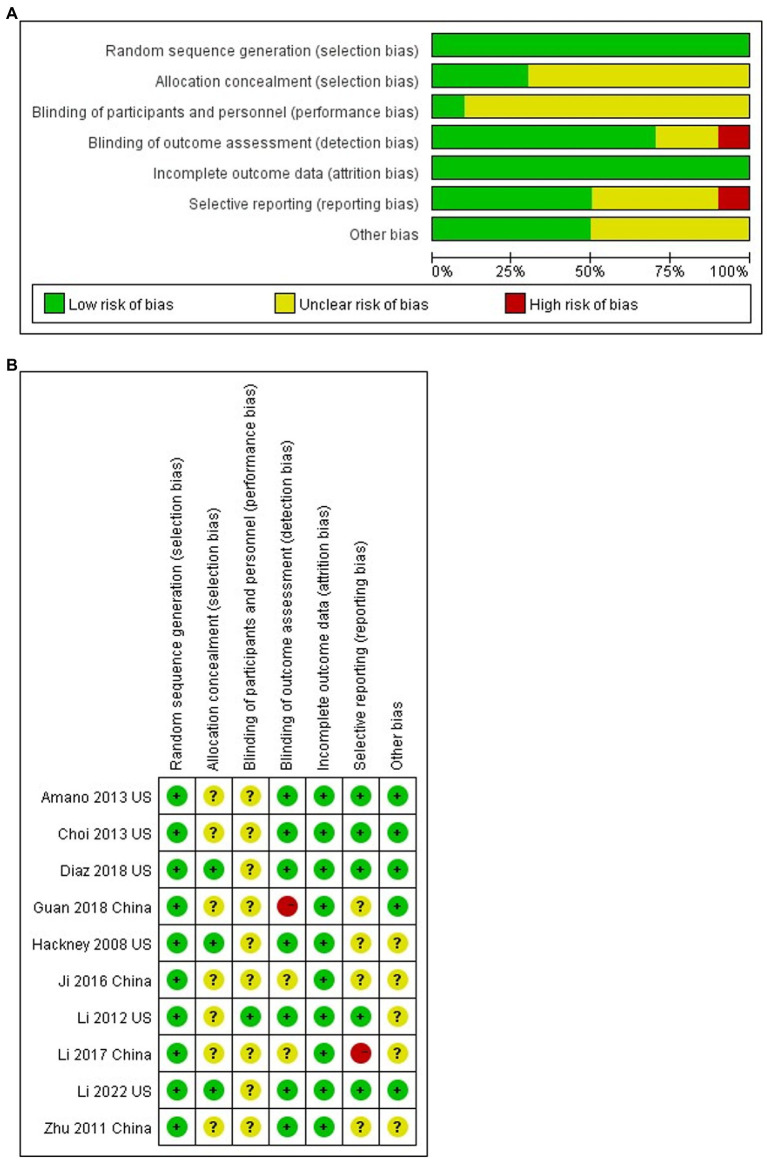
**(A)** Risk of bias graph. **(B)** Risk of bias summary.

Two investigators (ZPA and LZL) assessed study quality using the Cochrane risk of bias instrument: random sequence generation, allocation concealment, blinding (participants, therapists, and assessors), incomplete outcome data, selective reporting and other biases, with each item judged and categorized for study quality using low risk of bias, uncertain risk of bias and high risk of bias. The quality of the 10 studies included in this meta-analysis is shown in Figure 2.

### The effect of Tai Chi on motor function

**Figure 3:**
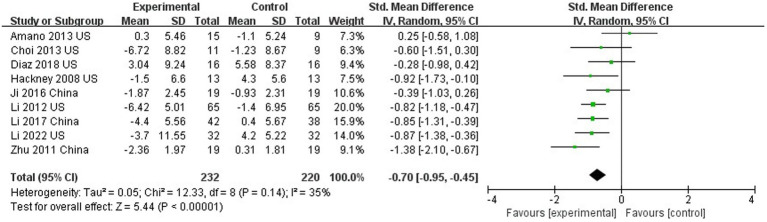
The effect of Tai Chi on motor function.

**Figure 4:**
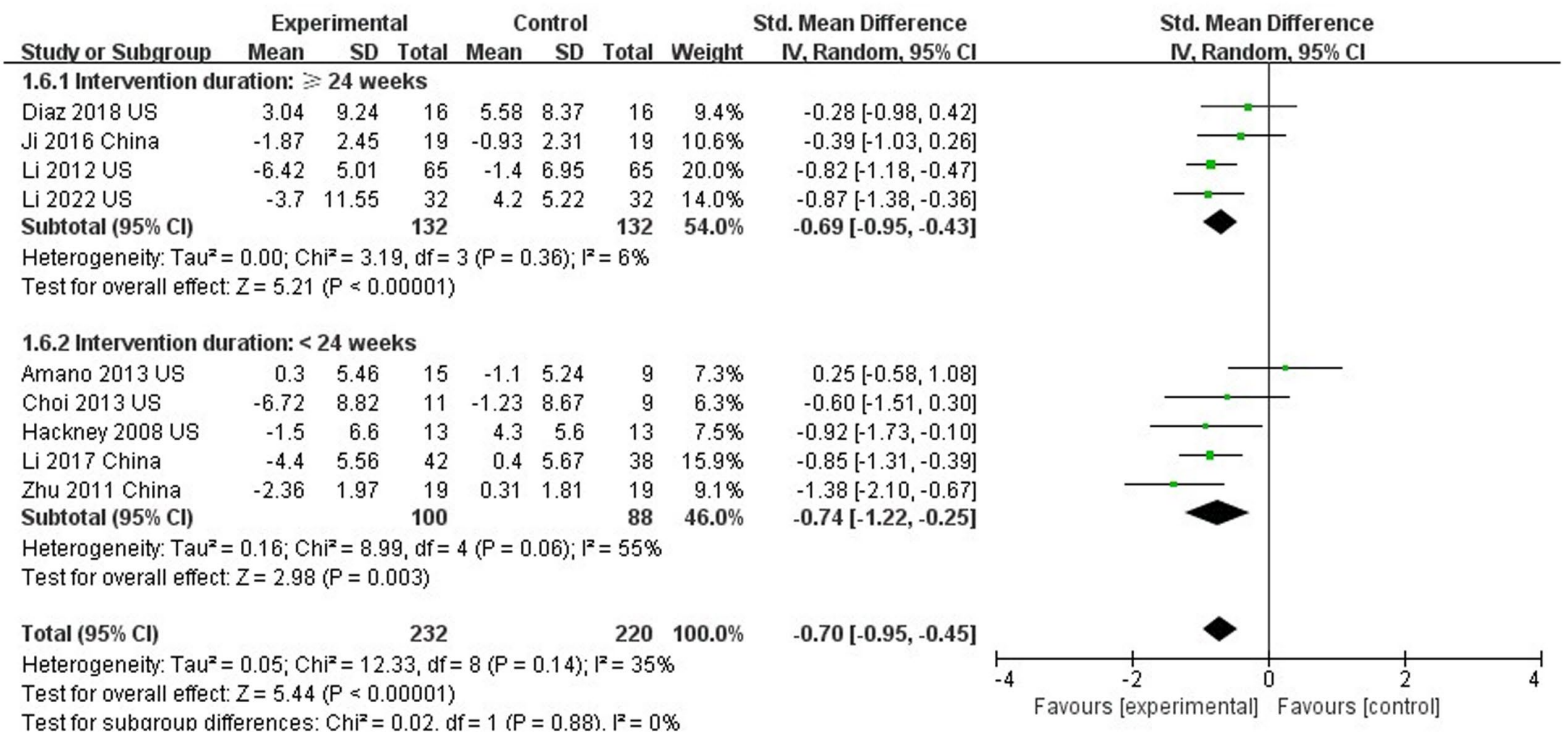
Influence of different intervention time on motor function (subgroup analysis).

Nine out of 10 studies assessed motor function using UPDRS III (Hackney and Earhart, 2008; Yi et al., 2011; Li et al., 2012, 2017, 2022; Amano et al., 2013; Choi et al., 2013; Suqiong et al., 2016; Vergara-Diaz et al., 2018). The results showed that TC was able to improve the motor function of PD patients compared with the control group (SMD = −0.70; 95%CI = −0.95, −0.45; *p* < 0.001; *I*^2^ = 35%; Figure 3). This meta-analysis sought to analyze the effect of intervention duration of TC on motor function in PD patients, and the results showed that no significant difference between the intervention durations (≥24 weeks compared to <24 weeks; SMD = −0.70; 95%CI = −0.95, −0.45; *p* = 0.88; *I*^2^ = 0%; Figure 4).

### The effect of Tai Chi on balance

**Figure 5:**
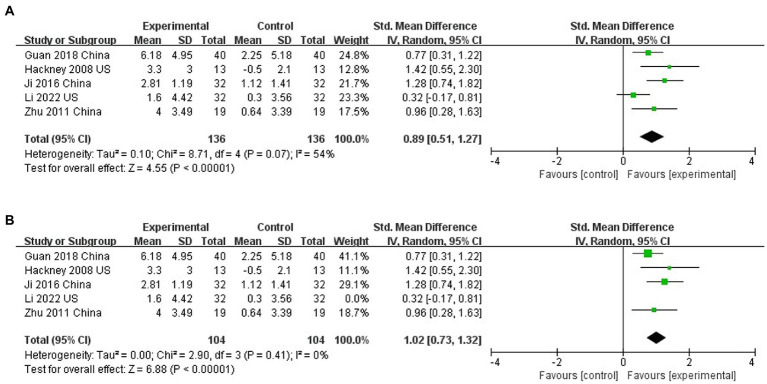
**(A)** The effect of Tai Chi on balance. **(B)** The effect of Tai Chi on balance (Sensitivity analysis).

Five studies assessed the effects of TC on balance function using the BBS (Hackney and Earhart, 2008;Yi et al., 2011;Suqiong et al., 2016; Xihong et al., 2018; Li et al., 2022). TC significantly improved balance function compared to controls (SMD = 0.89; 95% CI = 0.51, 1.27; *p* < 0.001; *I*^2^ = 54%; Figure 5A). Sensitivity analysis one by one found that only by excluding the study of Li et al. (2022), the heterogeneity would be significantly reduced, but the results did not change (SMD = 1.02; 95%CI = 0.73, 1.32; *P*<0.001; *I*^2^ = 0%; Figure 5B). This phenomenon shows that the research of Li et al. (2022) is the main reason for high heterogeneity.

### The effect of Tai Chi on functional walking

**Figure 6:**
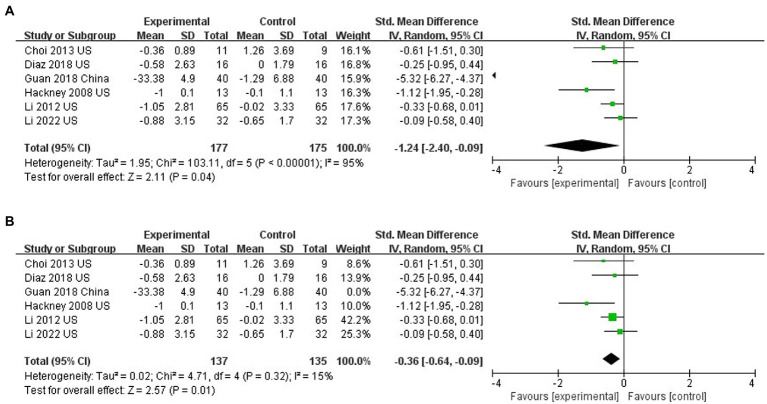
**(A)** Effects of Tai Chi on walking ability (TUG). **(B)** Influence of Tai Chi on Walking Ability (TUG; Sensitivity analysis).

Six studies assessed the functional walking of PD patients with TUG (Hackney and Earhart, 2008; Li et al., 2012, 2022 Choi et al., 2013; Vergara-Diaz et al., 2018; Xihong et al., 2018;). TC improved functional walking compared to controls (SMD = −1.24; 95% CI = −2.40, −0.09; *p* = 0.04; *I*^2^ = 95%; Figure 6A). However, there was statistically significant heterogeneity between studies (*I*^2^ = 95%) with one study visually heterogeneous from others (Xihong et al., 2018). The sensitivity analysis excluding the study by Xihong et al. (2018), results were still statistically different, and the heterogeneity was obviously reduced (SMD = −0.36; 95% CI = −0.64, −0.09; *p* = 0.01; *I*^2^ = 15%; Figure 6B).

### The effect of Tai Chi on walking endurance

**Figure 7:**
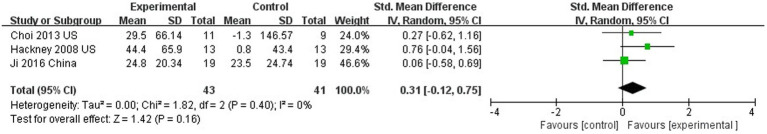
The effect of Tai Chi on walking (6MWT).

Three studies evaluated the walking endurance of PD patients using the 6MWT (Hackney and Earhart, 2008; Choi et al., 2013; Suqiong et al., 2016). TC did not improve walking endurance compared to controls (SMD = 0.31; 95% CI = −0.12, 0.75; *p* = 0.16; *I*^2^ = 0%; Figure 7).

### The effect of Tai Chi on gait

**Figure 8:**
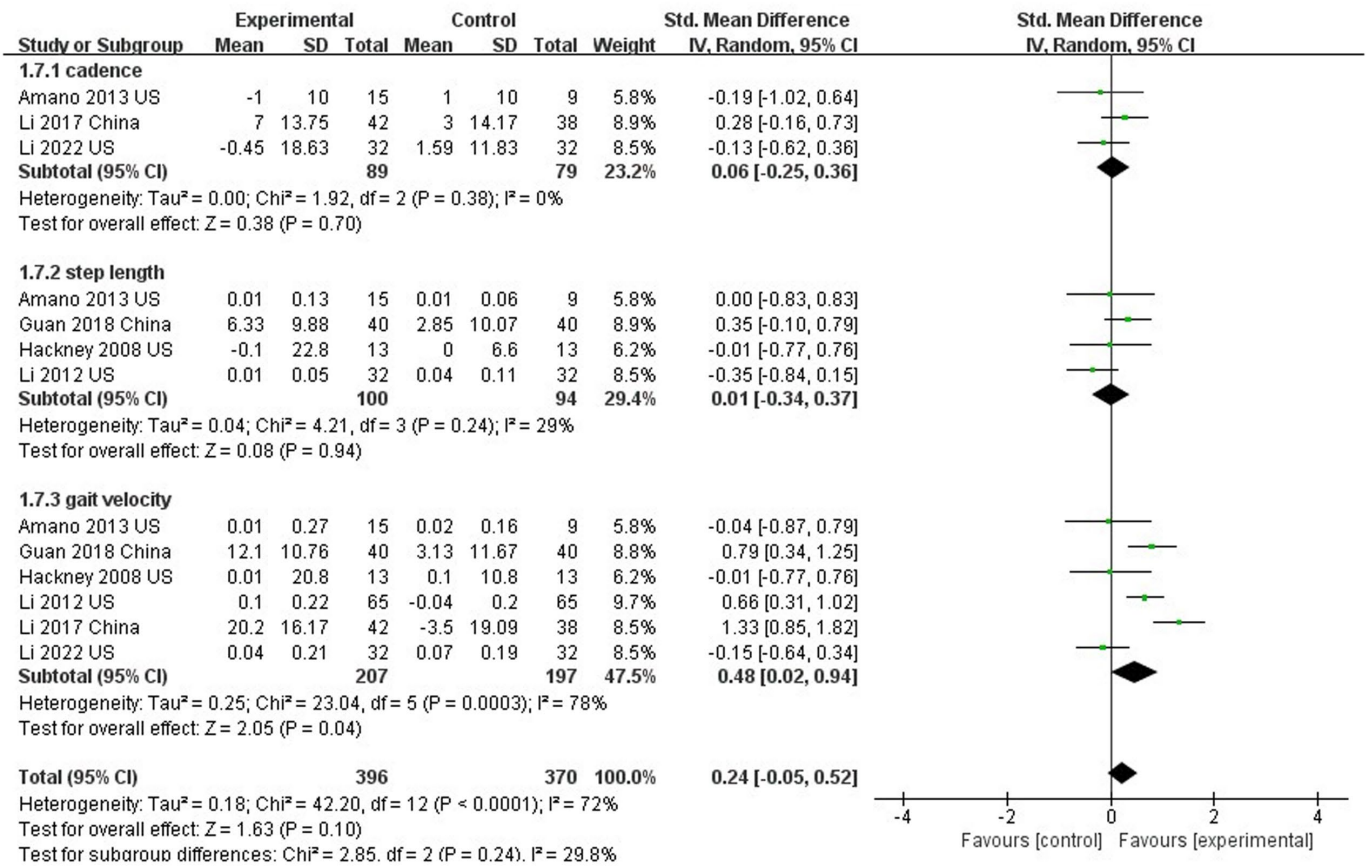
The effect of Tai Chi on walking (Gait).

Six studies evaluated gait in PD patients (Hackney and Earhart, 2008; Li et al., 2012, 2017, 2022; Amano et al., 2013; Xihong et al., 2018). In this analysis, no significant differences between TC and control groups were found in cadence (SMD = 0.06; 95% CI = −0.25, 0.36; *p* = 0.70; *I*^2^ = 0%; Figure 8) or stride length (SMD = 0.01; 95% CI = −0.34, 0.37; *p* = 0.94; *I*^2^ = 29%; Figure 8), but there was significant improvement in gait velocity (SMD = 0.48; 95% CI = −0.02, 0.94; *p* = 0.04; *I*^2^ = 78%; Figure 8).

## Discussion

This systematic review and meta-analysis showed that in addition to gait velocity improvement, TC also able to make significantly improved motor function, balance, and functional walking that were assessed with UPDRS III, BBS, and TUG, respectively. However, no significant differences were identified in endurance, stride length, and cadence.

### General motor function

As a traditional Chinese psychosomatic therapy, TC has become a complementary and alternative therapy for patients with PD (Song et al., 2017). TC is characterized by light, slow movements of the mind that focus on body posture, muscle tone, and breathing with attentive consciousness (Wu, 2002). TC requires subjects to perform dynamic movements, which may affect gait, balance, and other functional activities (Vergara-Diaz et al., 2018). TC may have greater benefits for patients with PD compared with regular exercise. TC involves multifaceted training (balance, flexibility as well as neuromuscular coordination training) that, at the same time, incorporates a number of cognitive components during training, such as body awareness, focused attention, imagery, etc., which improves PD motor abilities by performing planned goal-directed training (Wayne, 2013).

According to previous studies, dyskinesia, bradykinesia, hypokinesia, postural instability, rigidity, bending posture, and tremor at rest are the main signs and symptoms of PD patients (de Lau and Breteler, 2006; Teive et al., 2016; Lotankar et al., 2017). UPDRS III is the most widely used measure of in clinical and research assessment of motor function in PD patients (Siderowf et al., 2002).

Our study found that PD patients’ motor function (especially gait velocity and balance) improved significantly after TC training, which is consistent with the results of previous studies (Li et al., 2012; Li and Harmer, 2015). A previous meta-analysis showed that TC could improve UPDRS III scores and motor function in PD patients (Yang et al., 2014). To explore the effect of long-term TC exercise on the motor symptoms of PD patients and its potential mechanism, Li et al. (2022) conducted a year-long study and concluded that long-term TC training can improve motor function, especially gait and balance function, in PD patients. Huntingtin interaction protein 2 (HIP2) is an E2 ubiquitin-conjugating enzyme associated with neurodegenerative diseases. One study found HIP2 mRNA levels increase significantly in PD patients after TC training (Li et al., 2022). In addition, there are also studies that show that Decreased expression of HIP2 has been reported in the blood and the substantia nigra of PD patients (Grünblatt et al., 2004; Scherzer et al., 2007; Karlsson et al., 2013). In PD models, decreased HIP2 expression leads to impaired motor function and increased vulnerability to dopaminergic degeneration (Su et al., 2018). These evidences confirm that TC training can reverse the downregulation of HIP2 mRNA, and this change is related to the improvement of motor function in PD patients, suggesting that Tai Chi training can decrease the vulnerability to dopaminergic degeneration in PD.

With the 24-week as the demarcation mark, this present study found that TC is able to improve the motor function of patients with PD regardless of more or less than 24 weeks. However, there is no obvious difference when comparing the improvements between less and more than 24-week studies. Of the studies included in this meta-analysis, only 4 studies were ≥ 24 weeks and 5 studies were<24 weeks. Currently, there is insufficient evidence to determine the optimal TC intervention time. Therefore, longer duration of TC study may be needed in the future to determine the effect of long-term TC intervention.

### Balance (BBS and TUG)

A Meta-analysis study on inflammatory cytokines in PD showed that interleukin-1β (IL-1β) levels were significantly elevated in blood (Qin et al., 2016). In addition, it has been found that IL-1β induced PD symptoms in wild-type animals stimulated by lipopolysaccharide (LPS), suggesting that IL-1β may contribute to the occurrence or progression of PD (Saghazadeh et al., 2016). TC can improve the BBS of PD patients, which may be related to the down-regulation of proinflammatory cytokines, especially downregulation of IL-1β (Li et al., 2022). Previous studies found that PD patients with freezing of gait showed reduced network connectivity in the visual network (VN; Rogge et al., 2018; Ruan et al., 2020). The VN is composed of bilateral striate and extrastriata visual Areas (Pedersini et al., 2020). Visual proprioceptive conflict may affect gait and balance, and visual cues can improve balance and prevent falls in patients with PD (Assländer et al., 2015). There are also fMRI studies showing that changes in BBS scores are associated with changes in VN (Li et al., 2022). Whereas TC training can enhance VN connectivity (Li et al., 2022), therefore, the ability of TC to improve BBS may also be associated with enhanced VN function.

TC has been used to enhance postural control, especially for those suffering from complicated conditions with disruptions in their visual and somatosensory systems (Lin et al., 2000; Wong et al., 2001). The practice of TC is thought to increase awareness of body alignment during movement by focusing on the placement of the feet, an upright position of the head and trunk, and the intentional, attentive body movement in the direction of the specific TC postures (Liu and Frank, 2010). Furthermore, several scientific studies of TC have demonstrated improvements in lower extremity range of motion (Wu et al., 2004), strength (Wu et al., 2004), and proprioception (Xu et al., 2004), as well as enhanced neuromuscular responses involved in controlling the ankle joint during perturbations (Gatts and Woollacott, 2006) and in controlling stepping strategies of the swing leg during gait (Gatts and Woollacott, 2007). These functional improvements may also potentially contribute to the improvement of balance. Liu and Frank (2010) reviewed a number of studies in which balance function did not progress after TC training and concluded that for TC practice to achieve the effect of improving balance, the TC protocol needs to last for 12 weeks or more, with a frequency of at least 2 times per week and at least 45 min of TC meeting time. Of the five studies included in the analysis, only one lasted 4 weeks (Yi et al., 2011); the remaining four (Hackney and Earhart, 2008; Lan et al., 2008; Suqiong et al., 2016; Xihong et al., 2018; Li et al., 2022) were all 12 weeks or longer. In terms of frequency, the frequency was at least twice a week in four of the five studies (Hackney and Earhart, 2008; Yi et al., 2011; Lorefice et al., 2017; Xihong et al., 2018; Li et al., 2022) and once a week in one study (Suqiong et al., 2016). In terms of TC per intervention time, four studies (Hackney and Earhart, 2008; Shu et al., 2014; Suqiong et al., 2016; Xihong et al., 2018; Li et al., 2022) exceeded 45 min and one study was less than 45 min (Yi et al., 2011). Most of the studies we included in the analysis met the TC protocol required by Liu and Frank (2010), therefore, this may also be one of the reasons for our positive results in terms of balance.

The results of this study also showed a significant improvement in TUG in PD patients after TC training. TUG test has been used to assess balance and mobility in Parkinson’s Disease (Podsiadlo and Richardson, 1991; Zampieri et al., 2010). The TUG demands appropriate initiation of stepping, acceleration and deceleration, and preparation to turn twice. The process of first turn and last turn to sit down is challenging for PD patients (Nordin et al., 2006). There are studies testing the use of the TUG test for quantitative fall risk assessment, and the results show that the TUG is able to effectively assess fall risk (Greene et al., 2010). At the same time, TUG is also able to evaluate walking/coordination related tasks, as well as mobility related tasks (Lorefice et al., 2017), and previous studies have confirmed the validity of the tug test as a measure of functional mobility (Sebastião et al., 2016). These evidences suggested that the improvement of TUG also represented the improvement of balance function as well as mobility to some extent. PD patients in the present meta-analysis had positive results in terms of both BBS and tug after training with TC. This lends credence to our findings (TC was able to improve patients’ balance function and mobility).

### Endurance

This study concluded that TC training was able to improve TUG, and could not improve endurance in walking. This is consistent with a previous findings (Yang et al., 2014). Poor performance at the 6MWT may be related to cardiopulmonary function in PD patients, and some studies considered that many PD participants had poor cardiopulmonary function and insufficient levels of physical activity, which may affect 6MWT performance (Garber and Friedman, 2003). A previous study also yielded a negative result, and analysis found that all enrolled patients had subnormal 6MWT results, suggesting that poor cardiopulmonary function may be the cause of poor endurance performance (Choi et al., 2013). However, some studies have suggested that TC can improve the endurance performance of PD patients (Li et al., 2022). A meta-analysis showed aerobic exercise was able to improve endurance performance in PD patients (Shu et al., 2014). TC is generally 40 min or more per session and can achieve mild to moderate intensity aerobic exercise (Lan et al., 2008), which may be responsible for TC’s ability to improve endurance performance in PD patients. At present, there is no exact result on whether TC improvement improves endurance in PD patients.

### Gait parameters

Six studies in this meta-analysis evaluated gait, and the combined effect showed no significant improvement in cadence or stride length with TC training compared with controls, but a beneficial effect in gait velocity. A meta-analysis exploring the effect of TC on gait in PD patients showed no improvement in step size or gait speed with TC training compared to controls (Yang et al., 2014), but it included only two studies. Furthermore, Shinichi Amano et al. also found that 16 weeks of TC was not effective in improving gait initiation or gait performance in patients with Parkinson’s disease (Amano et al., 2013). TC as a self-controlled, gentle balance perturbation exercise, it does not require high speed during exercise, and emphasizes soft, light, and slow movements. TC trains body stability and mobility from form to form on lower limbs on different surfaces of support through solid/empty stances, and perturbates the body’s center of gravity through a variety of opening/closing or defensive/offensive TC movements and postures (Liu and Frank, 2010). TC training focuses more on the stability and flexibility of the lower limbs, and does not pay much attention to gait. Although the present study yielded inconsistent results with previous studies, the 6 included studies had high heterogeneity. There was no robust evidence to support that TC has a beneficial effect in gait.

## Limitations

This systematic review has several limitations. First, we did not conduct further analyses on the style of TC because three studies did not specify what style was, and second, the TC style of the majority of included studies was yang style, making it impossible to judge which style worked better with treatment. Second, none of the RCTs included in this review did not specify the different PD subtypes, so the effect of TC on different subtypes of PD is uncertain. Finally, because of the small number of eligible studies, only subgroup analysis of motor function was performed, and no subgroup analysis of other outcome measures was performed.

## Conclusion

The meta-analysis showed that TC improved motor function, balance function, functional walking capacity, as well as gait speed in PD patients, but had no effect on walking endurance, stride length, as well as cadence. Due to its holistic approach and multi-benefits to people who have been practicing TC, we surely recommend TC to any patients who want to give a try.

## Author contributions

XB and Y-xL proposed the original idea and design. HL and LB provided valuable revision suggestions for the completion of the manuscript. P-aZ wrote the manuscript. P-aZ, Q-qL, and Z-lL conducted the search, screening, and data analysis. R-lH and SX completed the data extraction. All authors contributed to the article and approved the submitted version.

## Conflict of interest

The authors declare that the research was conducted in the absence of any commercial or financial relationships that could be construed as a potential conflict of interest.

## Publisher’s note

All claims expressed in this article are solely those of the authors and do not necessarily represent those of their affiliated organizations, or those of the publisher, the editors and the reviewers. Any product that may be evaluated in this article, or claim that may be made by its manufacturer, is not guaranteed or endorsed by the publisher.
